# Physicians' perceptions of an electronic health record-based clinical trial alert approach to subject recruitment: A survey

**DOI:** 10.1186/1472-6947-8-13

**Published:** 2008-04-02

**Authors:** Peter J Embi, Anil Jain, C Martin Harris

**Affiliations:** 1 Center for Health Informatics and Department of Medicine, University of Cincinnati Academic Health Center, 231 Albert Sabin Way, PO Box 670840, Cincinnati, Ohio, USA; 2 Divisions of Information Technology and Medicine, Cleveland Clinic Foundation, 9500 Euclid Ave, Cleveland, Ohio, USA

## Abstract

**Background:**

Physician participation in clinical research recruitment efforts is critical to many studies' success, but it is often limited. Use of an Electronic Health Record (EHR)-based, point-of-care Clinical Trial Alert (CTA) approach has led to significant increases in physician-generated recruitment and holds promise for wider benefit. However, little is known about physicians' decision-making regarding recruitment in EHR-equipped settings or the use of such EHR-based approaches. We sought to assess physicians' perceptions about recruitment in general and using the CTA approach in particular.

**Methods:**

We developed and delivered a Web-based survey consisting of 15 multiple-choice and free-text questions. Participants included the 114 physician subjects (10 endocrinologists and 104 general internists) who were exposed to CTAs during our preceding 4-month intervention study. Response data were descriptively analyzed, and key findings were compared between groups using appropriate statistical tests.

**Results:**

Sixty-nine physicians (61%) responded during the 10-week survey period. Respondents and non-respondents did not differ significantly. Twenty-seven percent of respondents felt very comfortable recruiting patients to trials in general, and 77% appreciated being reminded about a trial via a CTA. Only 11% percent felt the CTA was difficult to use, and 27% felt it was more than somewhat intrusive. Among those who ignored all CTAs, 37% cited a lack of time, 28% knowledge of the patient's ineligibility, and 13% limited knowledge about the trial as their most common reason. Thirty-eight percent wanted more information about the trial presented in the CTA, and 73% were interested in seeing CTAs for future trials. Comments and suggestions were submitted by 33% of respondents and included suggestions for improvement of the CTA approach.

**Conclusion:**

Most physicians were comfortable recruiting patients for clinical trials at the point-of-care, found the EHR-based CTA approach useful and would like to see it used in the future. These findings provide insight into the perceived utility of this EHR-based approach to subject recruitment, suggest ways it might be improved, and add to the limited body of knowledge regarding physicians' attitudes toward clinical trial recruitment in EHR-equipped settings.

## Background

Physician participation is critical to the successes of most clinical trial recruitment efforts. Not only do clinicians play a vital role by identifying potentially eligible subjects, but patients are much more likely to participate in a study if their physician has suggested it to them [1]. Unfortunately, the identification and recruitment of eligible patients during the course of busy clinical practice can be difficult. In order to successfully recruit patients, physicians engaging in traditional recruitment have to remember which local clinical trials are active, recall the trial's details in order to determine patient eligibility, take time to explain the trial's details to potentially eligible patients, and often take more time to perform other recruitment activities. Doing all of this while also attempting to provide the individual patient with good care during a short clinic visit can be difficult, at best. Current privacy regulations add further challenges to overcome in solving this problem [2].

As a consequence, few clinicians, mostly those in university settings, do most of the recruiting for clinical trials [1,3,4]. Even in fields like oncology where clinical trial participation is considered optimal for many patients, only about 3% of eligible patients are enrolled into clinical trials and enrollment is often not representative of the general population [3,5,6]. In addition to frustrating progress, these factors can introduce bias to the trial and prevent some patients from receiving potentially beneficial 'state-of-the-art' therapy.

Numerous technological approaches have been developed in attempts to enhance clinical trial recruitment [7-14]. Some have shown promise by using computerized clinical databases to automate the identification of potentially eligible patients [15,16]. Electronic Health Record (EHR)-based approaches have also been described, though mostly in specialized settings and few have demonstrated significant benefit in controlled studies [17-20]. Until recently, whether comprehensive EHRs could be leveraged for the benefit of clinical trial recruitment at the point-of-care as effectively as they have been for patient safety and healthcare quality remained to be determined [21].

### The Clinical Trial Alert Approach

In 2004, we developed an EHR-based Clinical Trial Alert (CTA) approach. The approach was designed to overcome many of the known obstacles to trial recruitment by physicians while complying with current privacy regulations [22]. We conducted a before-after intervention study of the CTA approach applied to a large, multi-center, NIH-sponsored type 2 diabetes mellitus clinical trial, the Action to Control Cardiovascular Risk in Diabetes (ACCORD) study for which the Cleveland Clinic was a study site. During our 4- month CTA intervention phase, the system alerted physicians to patients whose EHR data met selected trial eligibility criteria [23]. Upon triggering, the CTA served to remind the clinician about the trial and to facilitate referral of interested patients to a clinical trial's coordinator. Our subjects for this study were the 114 staff physicians (10 endocrinologists, 104 general internists) practicing at one of our health system's Internal Medicine and Endocrinology referral-center-based and community-based clinics. When presented with the CTA, physicians could choose to ignore the CTA or to use it.

In our intervention study, CTA use resulted in significant increase in the number of physicians participating in recruitment activities and in their rates of subject referrals and enrollments to the trial compared to baseline rates [23]. During the intervention study, all 114 physician subjects received at least one CTA to which they could respond. From among those, 48 (42%) participated by attending to and processing at least one CTA order form while the remainder ignored all CTAs presented to them. Of those who participated by attending to at least one alert during the intervention phase, 42 (88%) referred at least one patient to the trial coordinator, and 11 (23%) generated at least one enrollment. The number of physicians referring patients after CTA activation increased more than eight-fold, from 5 before to 42 after (*P *< 0.001). In addition, physician-generated referral rates increased more than ten-fold, from 5.7/month before CTA activation to 59.5/month afterward (*P *< 0.001), and enrolment rates more than doubled, from 2.9/month before to 6.0/month after (*P *= 0.007). While general internists had not contributed to recruitment before, they generated 170 (71%) of the referrals and 7 (29%) of the enrollments after CTA activation. CTA use was also associated with a substantial referral rate increase of 47% among endocrinologists. Despite these improvements in overall physician recruitment to this trial after CTA activation, 52% of physicians did not use the CTA even once and nearly 90% of all CTAs triggered were ignored by physicians.

### Purpose for the Survey

While the preceding intervention study indicated that the CTA approach had a significant impact on recruitment and enrollment rates by physicians, it also revealed considerable inefficiencies. Moreover, the intervention study was not designed to answer certain important questions about physicians' attitudes and reasons for using or dismissing the EHR-based recruitment alerts. Despite prior research having been conducted in the areas of point-of-care recruitment and computerized clinical decision support, little is known about how physicians feel about point-of-care recruitment in EHR-equipped settings, and we know of no reports concerning their perceptions on using EHRs to facilitate point-of-care recruitment in the manner allowed by the CTA approach.

Therefore, we undertook a survey of those physicians who were exposed to CTAs during our recently completed intervention study. Our objective was to assess their perceptions of the CTA approach and of trial recruitment in general in order to better understand the findings of our intervention study and to inform further development, application, and evaluation of the EHR-based approaches to research subject recruitment.

## Methods

To develop our 15-question survey (Table 1), we drew upon factors identified in prior research studies to influence physician participation in trial recruitment and their use of similar EHR-based technologies. Questions were organized and delivered to participants using a commercial, Web-based survey service (SurveyMonkey.com).

**Table 1:** Survey Questions

**Questions**	**Possible responses**
1. Did you know about the existence of the ACCORD clinical trial at CCF prior to being presented with the ACCORD Clinical Trial Alert (CTA)?	__ Yes __ No

2. Did you appreciate being reminded during the patient encounter that your patient might be eligible for the ACCORD clinical trial?	__ Yes __ No

3. How did you respond when presented with the Clinical Trial Alert?	__ a. I chose "No" to Cancel the alert EVERY TIME __ b. I chose "No" to Cancel the alert SOMETIMES __ c. I always chose "Yes" in order to proceed to the CTA's Smart-Set __ d. I don't recall

4. If you ever chose "No" to cancel, please rank all reasons in order of how frequently they influenced your decision to disregard the CTA: (1-most common reason, 5-least common reason)	__ I didn't have time to respond __ I already knew my patient would not qualify for the study __ I didn't feel knowledgeable enough about the trial to discuss it __ I felt that discussing this trial would adversely impact the doctor-patient relationship __ Other: (please type your other reason(s)): _____

5. How comfortable are you discussing participation in clinical trials with your patients, in general?	1-------------2-------------3-------------4-------------5 Uncomfortable Somewhat Comfortable Very Comfortable

6. How comfortable were you asking your patient(s) to consider the participating in the ACCORD trial?	1-------------2-------------3-------------4-------------5 Uncomfortable Somewhat Comfortable Very Comfortable or __(I Never Asked)

7. Overall, how easy was the Clinical Trial Alert system to use?	1-------------2-------------3-------------4-------------5 Difficult Moderately Easy Very Easy

8. Overall, how intrusive did you find the Clinical Trial Alert(s) to be during the patient care?	1-------------2-------------3-------------4-------------5 Not Intrusive Somewhat Intrusive Very Intrusive

9. Overall, did you find you wanted more information about the trial provided with the alert?	__ Yes, the information provided was NOT adequate __ No, the information provided was adequate

10. How interested would you be in seeing such alerts for future clinical trials?	1-------------2-------------3-------------4-------------5 Not Interested Somewhat Interested Very Interested

11. (part 1) If these alerts were improved so that they appeared only if a patient were definitely eligible for a clinical trial, how interested would you be in receiving such alerts for future trials?	1-------------2-------------3-------------4-------------5 Not Interested Somewhat Interested Very Interested
11. (part 2) If still not interested, why not?	__ a. I'm too busy to participate in such activities __ b. I don't want to discuss clinical trial participation with my patient __ c. Other: (please specify)______

12. If your patient were eligible for more than one ongoing clinical trial, would you be interested in receiving an alert about each of them, only one, or none?	__ a. Each – an alert for each trial and I will choose __ b. Only one – an alert for the trial that is probably the best fit for my patient __ c. None – I don't want to see any alerts about clinical trials for which my patient may be eligible

13. Is this a technology that you would like to use for your future trials?	__ a. Yes, I would like to use CTAs for my future clinical trials __ b. No, I would prefer to use only traditional methods of recruitment __ c. I do not conduct clinical trials

14. Please provide at least one suggestion for improving the CTA in the future?	(free text response)

15. Please provide any additional comments you wish:	(free text response)

Participation in the IRB approved survey was solicited via email from all physicians who were targeted for the CTA intervention. These included the 10 endocrinologists and 104 general internists on staff at the main campus and community based endocrinology and general internal medicine clinics of the Cleveland Clinic who had been exposed to the CTA at least once over the prior 4-month intervention study. Among the endocrinologist subjects, one was the site Principal Investigator for the clinical trial to which the CTA intervention was applied.

Physician subjects could respond to the Web-based survey or to an email version. Up to three reminders were sent to those who had not responded over the 10-week survey period. Responses were transferred onto a computerized spreadsheet. Response frequencies were calculated and the data were descriptively analyzed. Differences between groups over relevant variables were tested using Fisher's exact test. Narrative comments were coded and categorized independently by two authors and agreement on final categorizations was finally achieved via an iterative discussion process.

## Results

Sixty-nine physicians (61%) responded during the 10-week survey period. All but seven responded via the Web-based survey instrument, with the rest responding via Email. A greater proportion of endocrinologists (90%) than internists (58%) responded, *P *= 0.09. Respondents were not significantly more likely to be CTA users (58%) than non-users (42%), *P *= 0.13. Even among respondents who opted to review CTAs when presented rather than simply dismiss them (i.e. CTA users), many did not encounter a patient suitable for referral. Indeed, nearly half (48%) of all survey respondents did not use the CTA to refer any patients during our study.

### General Recruitment Issues

**Figure 1:**
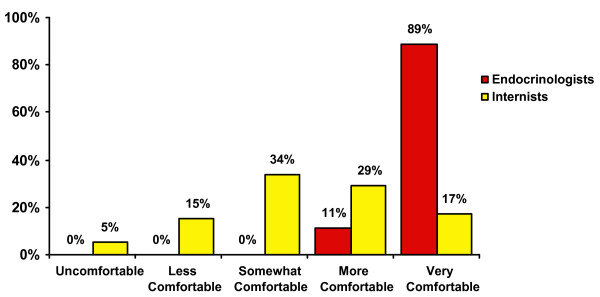
Level of comfort with clinical trial recruitment by degree of specialization.

Most respondents (83%) felt at least *somewhat comfortable *discussing participation in any clinical trial with 27% feeling *very comfortable*. Endocrinologists and general internists varied in their levels of comfort (Figure 1). Respondents were slightly more comfortable discussing participation in the associated type 2 diabetes mellitus clinical trial that was the focus of our CTA intervention. Overall, 31% felt *very comfortable *recruiting for this particular trial, 100% of endocrinologists and 20% of internists. Thirty-two percent of respondents reported not knowing about the diabetes trial prior to CTA activation despite being exposed to traditional efforts to inform them of the trial and encourage them to participate (e.g. emails, discussion at meetings, posted information about the trial in the clinic setting).

### Use and perceptions of the CTA approach

**Figure 2:**
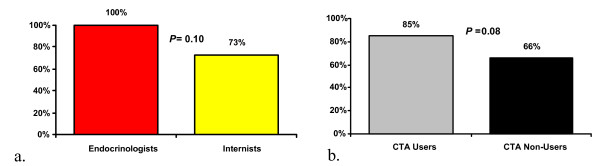
Percentage who appreciated being reminded of the CTA approach by (a) specialty and (b) CTA user status.

Most respondents (77%) appreciated being reminded about the trial via CTA during the patient encounter. Though high even among subgroups, level of appreciation varied somewhat by degree of specialization as well as between CTA-users and CTA non-users (Figure 2). Despite these levels of appreciation, 79% stated that they dismissed CTAs sometimes (54%) or every time (25%), with only 9% indicating they used the CTA to consider patients for referral every time it appeared. These reported rates were consistent with those observed by direct query of the system during the preceding intervention study. When asked to rank their reasons for dismissing a CTA, 37% cited a lack of time, 28% cited knowledge that the patient would not qualify, 13% cited limited knowledge about the trial, and only 4% cited concern about adversely impacting the doctor-patient relationship as their most common reason. Overall, 38% wanted the CTA to contain more information about the associated clinical trial than it did. This included 11% of endocrinologists versus 43% of general internists (*P *= 0.13), and 31% of CTA Users versus 50% of CTA Non-users (*P *= 0.13). Those who indicated "lack of time" as their top reason for dismissing alerts were also split with 50% indicating that they wanted the CTA to contain more information. Only 11% felt the CTA was *difficult *to use; 27% felt it was more than just *somewhat intrusive*.

**Figure 3:**
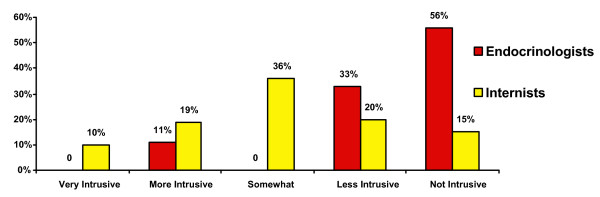
Perceived intrusiveness of the CTA approach by specialty.

**Figure 4:**
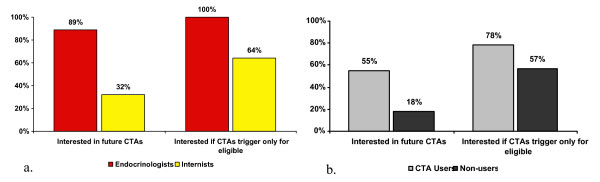
Interest in using an EHR-based CTA in the future by (a) specialty and (b) CTA user status.

Endocrinologists generally found the CTA approach less intrusive than did general internists (Figure 3). Seventy-three percent were interested in being presented with CTAs for future trials; this rose overall to 87% if future CTAs were designed to trigger only for patients with a high likelihood of eligibility for the trial, and it varied by degree of specialization and between CTA Users versus CTA Non-users (Figure 4). Among the 20% who did not want the CTA even if it were made more specific, 71% indicated time constraints to be their main reason. Among those who wanted to be presented with CTAs in the future, 42% wanted an alert for each trial for which a patient may qualify, while 58% wanted a CTA for just the one "best" trial. Among the 59% percent of respondents who indicated that they conduct clinical research, 88% indicated that they would like to use CTAs for their trials.

### Respondents' Comments

Narrative comments were submitted by 23 respondents (33%) and included suggestions for improving the CTA's content, appearance, or operation. Upon review and analysis, comments grouped into the categories presented in Table 2.

**Table 2:** Categories and Frequencies of Narrative Comments provided by 23 respondents

**Comment Category**	**Frequency**
Want more information about the trial in the CTA or via a link	43%
Increase the specificity of the CTA	13%
Generally positive comment	13%
Present the CTA at only one encounter per patient, then never again	9%
Simplify the onscreen interface	4%
Provide ability to opt-out	4%
Prefer a passive notification (i.e. not pop-up)	4%
Concerned about being overloaded by many alerts	4%
Disagree with the approach of recruiting patients at the point-of-care	4%

## Discussion

As EHRs are implemented in practices across the nation, their use for secondary purposes such as clinical research will undoubtedly increase. One approach that has already demonstrated the ability to help overcome the major clinical research obstacle of subject recruitment is the CTA approach. While this approach holds promise for improving the quality and efficiency of clinical research, its ultimate utility at the point-of-care rests on understanding how to make such an approach acceptable to and useful for clinicians.

As these survey results demonstrate, most internists exposed to the CTA during our initial intervention study were generally agreeable to this approach to trial recruitment, even at this early stage in its design. Perhaps not surprisingly, there were some differences between generalists and subspecialists. Overall, subspecialists were more comfortable with trial recruitment in general. Perhaps related to this, subspecialists were somewhat more positive than were generalist users regarding the CTA approach, finding it less intrusive and being a bit more appreciative of it. However, it is notable that most general internists and even a substantial minority of respondents who chose to ignore the CTA altogether still reported being appreciative of the approach and desired seeing it in the future. Speaking to this in another way is the fact that a surprisingly large proportion of respondents wished to receive an alert for each trial for which a future patient might qualify. While one might question whether these respondents would really want that if their patient qualified for a dozen trials ongoing in their region, and while such an approach would likely be suboptimal with regard to performance, this finding does appear to indicate an acceptance of this approach.

Of course, there was also a substantial proportion of respondents who found flaws with the current CTA approach. These are perhaps the most useful findings with regard to next steps in refining this approach. While it was our goal in designing the CTA approach to limit the information presented to clinicians and their need to discuss the trial in detail with their patients prior to study coordinator referral for full screening, it is clear that many wished to have more information about the trial available to them. Indeed, the finding that 50% of CTA non-users wanted more information about the trial seems to support the notion that providing such information might improve responsiveness to the CTA. Furthermore, as opposed to the challenges in overcoming some limiting factors such as lack of time to respond, modifying the CTA to provide a link to additional trial information is certainly a feasible solution to one identified limitation of our initial CTA approach. Another factor influencing CTA effectiveness relates to the balance between sensitivity and specificity of alerts that must be carefully weighed in future applications of this approach. What appears clear from both our intervention study and this survey is that targeting the CTA more specifically in order to avoid a high number of false-positive alerts would be welcomed by most users and might lead to improved CTA usage. Each of these issues is the focus of ongoing study.

This study has some limitations. While our 61% response rate is good for a physician survey and no significant difference in major characteristics of responders versus non-responders was found, it nevertheless is possible that non-responders may hold different views that would alter the results. Also, while the response rate from subspecialists was quite good, the limited number of subspecialists and the fact that they represent only one subspecialty mean that we must be cautious not to over-conclude with regard to the differences noted between subspecialists and generalists in this survey. Finally, as with our intervention study, these findings represent the perceptions of a limited group of practitioners from one health system, using the CTA approach in a single EHR platform, as applied to a single clinical trial. Therefore, while informative to those pursuing this approach in other settings, the generalizability of these findings remains to be determined.

## Conclusion

Most physicians felt that the CTA approach to point-of-care trial recruitment was easy to use and would like to see it used in the future, though subspecialists and generalists differed somewhat in their perceptions and many would like to see changes made to the CTA approach. These findings should help to inform future applications and refinement of this EHR-based point-of-care recruitment approach, and they add to the limited body of knowledge regarding physicians' attitudes toward clinical trial recruitment in EHR-equipped environments.

## Competing interests

The author(s) declare that they have no competing interests.

## Authors' contributions

PJE conceived the study, designed and conducted the survey, analyzed the results, and was the primary author of the manuscript; AJ participated in the survey design and analysis, and helped edit the manuscript; CMH participated in the survey design and helped support the study; all authors read and approved the final manuscript.

## Pre-publication history

The pre-publication history for this paper can be accessed here:



## References

[B1] Siminoff LA, Zhang A, Colabianchi N, Sturm CM, Shen Q (2000). Factors that predict the referral of breast cancer patients onto clinical trials by their surgeons and medical oncologists. J Clin Oncol.

[B2] U.S. Health Insurance Portability and Accountability Act of 1966. http://www.hhs.gov/ocr/hipaa/.

[B3] Winn RJ (1994). Obstacles to the accrual of patients to clinical trials in the community setting. Semin Oncol.

[B4] Taylor KM, Margolese RG, Soskolne CL (1984). Physicians' reasons for not entering eligible patients in a randomized clinical trial of surgery for breast cancer. N Engl J Med.

[B5] Schain WS (1994). Barriers to clinical trials. Part II: Knowledge and attitudes of potential participants. Cancer.

[B6] Somkin CP, Altschuler A, Ackerson L, Geiger AM, Greene SM, Mouchawar J, Holup J, Fehrenbacher L, Nelson A, Glass A (2005). Organizational barriers to physician participation in cancer clinical trials. Am J Manag Care.

[B7] Breitfeld PP, Weisburd M, Overhage JM, Sledge G, Tierney WM (1999). Pilot study of a point-of-use decision support tool for cancer clinical trials eligibility. J Am Med Inform Assoc.

[B8] Seroussi B, Bouaud J (2003). Using OncoDoc as a computer-based eligibility screening system to improve accrual onto breast cancer clinical trials. Artif Intell Med.

[B9] Ash N, Ogunyemi O, Zeng Q, Ohno-Machado L (2001). Finding appropriate clinical trials: evaluating encoded eligibility criteria with incomplete data. Proc AMIA Symp.

[B10] Papaconstantinou C, Theocharous G, Mahadevan S (1998). An expert system for assigning patients into clinical trials based on Bayesian networks. J Med Syst.

[B11] Thompson DS, Oberteuffer R, Dorman T (2003). Sepsis alert and diagnostic system: integrating clinical systems to enhance study coordinator efficiency. Comput Inform Nurs.

[B12] Ohno-Machado L, Wang SJ, Mar P, Boxwala AA (1999). Decision support for clinical trial eligibility determination in breast cancer. Proc AMIA Symp.

[B13] Fink E, Kokku PK, Nikiforou S, Hall LO, Goldgof DB, Krischer JP (2004). Selection of patients for clinical trials: an interactive web-based system. Artif Intell Med.

[B14] Gennari JH, Sklar D, Silva J (2001). Cross-tool communication: from protocol authoring to eligibility determination. Proc AMIA Symp.

[B15] Butte AJ, Weinstein DA, Kohane IS (2000). Enrolling patients into clinical trials faster using RealTime Recuiting. Proc AMIA Symp.

[B16] Weiner DL, Butte AJ, Hibberd PL, Fleisher GR (2003). Computerized recruiting for clinical trials in real time. Ann Emerg Med.

[B17] Afrin LB, Oates JC, Boyd CK, Daniels MS (2003). Leveraging of open EMR architecture for clinical trial accrual. AMIA Annu Symp Proc.

[B18] Carlson RW, Tu SW, Lane NM, Lai TL, Kemper CA, Musen MA, Shortliffe EH (1995). Computer-based screening of patients with HIV/AIDS for clinical-trial eligibility. Online J Curr Clin Trials.

[B19] Moore TD, Hotz K, Christensen R, Litwak D, Meyer J, Baxter T, Thompson M, Ashbury F (2003). Integration of clinical trial decision rules in an electronic medical record (EMR) enhances patient accrual and facilitates data management, quality control and analysis. Proc Am Soc Clin Onc.

[B20] Musen MA, Carlson RW, Fagan LM, Deresinski SC, Shortliffe EH (1992). T-HELPER: automated support for community-based clinical research. Proc Annu Symp Comput Appl Med Care.

[B21] Hersh W (2004). Health care information technology: progress and barriers. JAMA.

[B22] Embi PJ, Jain A, Clark J, Harris CM (2005). Development of an electronic health record-based Clinical Trial Alert system to enhance recruitment at the point of care. AMIA Annu Symp Proc.

[B23] Embi PJ, Jain A, Clark J, Bizjack S, Hornung R, Harris CM (2005). Effect of a clinical trial alert system on physician participation in trial recruitment. Arch Intern Med.

